# Wide-Field Oxygen Permeability Measurement of Contact Lenses Using a Modified Polarographic Electrode Cell

**DOI:** 10.3390/s26051725

**Published:** 2026-03-09

**Authors:** Wen-Hong Tong, Jing Liu, Jae-Yeon Pyo, Ki-Choong Mah, Seung-Jin Oh, Jae-Young Jang

**Affiliations:** 1 Department of Optometry, Eulji University, 553 Sanseong-daero, Sujeong-gu, Seongnam-si 13135, Republic of Korea; tongwenhong@gmail.com; 2 International College of Optometry, Jining Polytechnic, 77 Jinyu Road, Jining 272007, China; liudaojingem@163.com; 3 Olens Gangnam Daechi Optical Center, 102-1, 2936 Nambusunhwan-ro, Gangnam-gu, Seoul 06291, Republic of Korea; p961316@naver.com; 4 Korean Vision Care Research Institute, 12-17, 14 Gosanja-ro, Seongdong-gu, Seoul 04750, Republic of Korea; kcmah9927@gmail.com; 5 Luce Vision Center, B1, 401 Teheran-ro, Gangnam-gu, Seoul 06162, Republic of Korea; 6 Department of Optometry, Jeonbuk Science College, 117 Chungjeong-ro, Jeongeup-si 56204, Republic of Korea

**Keywords:** oxygen permeability, contact lenses, wide-field measurement, polarographic electrode cell, ISO 18369, oxygen transmissibility, corneal oxygenation

## Abstract

Oxygen permeability (Dk) is a key parameter for evaluating the ability of contact lenses to supply oxygen to the cornea. Although the polarographic method has been standardized as a reference technique for Dk measurement, conventional polarographic electrode cells are limited to a narrow central measurement area of approximately 4 mm in diameter, which may not adequately represent oxygen transport under actual wearing conditions. In this study, a modified polarographic electrode cell enabling wide-field oxygen permeability measurement over an expanded central area with a diameter of 11 mm was developed and evaluated under ISO 18369 measurement conditions. The performance of the proposed system was evaluated by comparing its accuracy, repeatability, and relative error with those of a conventional polarographic electrode cell using plano hydrogel contact lens samples with different uniform thicknesses. The Dk values obtained using the modified measurement cell did not show a statistically significant difference compared to those measured with the conventional measurement cell (t = 2.682, *p* = 0.055), and the relative error between the two systems was 1.93%, meeting the ISO acceptance criteria for the development of a new testing method. These results demonstrate that wide-field Dk measurement can be achieved without compromising reliability, providing a more representative and ISO-compliant approach for contact lens oxygen permeability evaluation.

## 1. Introduction

Contact lenses provide several advantages over conventional spectacles, including a wider field of view, resistance to fogging, and reduced susceptibility to environmental and weather conditions [1]. Among the various physical properties of contact lenses, oxygen permeability (Dk) and oxygen transmissibility (Dk/t) are regarded as two of the most critical parameters governing corneal physiology during contact lens wear. Dk/t reflects the capacity of a contact lens to deliver oxygen to the cornea by accounting for both the intrinsic permeability of the lens material and its thickness [2,3]. Adequate oxygen delivery is essential for maintaining corneal metabolic activity and transparency, as the avascular cornea relies primarily on atmospheric oxygen supplied through the tear film [4,5]. Insufficient oxygen supply has long been associated with clinical complications such as corneal edema, epithelial microcysts, neovascularization, and hypoxia-related discomfort [4,5,6].

Over the past two decades, advances in contact lens materials—particularly the introduction and widespread adoption of silicone hydrogel lenses—have substantially increased oxygen transmissibility compared to conventional hydrogel formulations [7,8,9]. Silicone hydrogel materials are now the dominant platform in soft contact lens prescribing due to their superior oxygen permeability and improved physiological performance [7,8,9]. In parallel, material engineering approaches continue to focus on optimizing intrinsic oxygen permeability while maintaining mechanical stability and biocompatibility [10,11].

However, oxygen delivery to the cornea depends not only on material Dk but also on lens thickness and power distribution. Variations in lens thickness significantly influence oxygen transmissibility, and local geometric differences can alter the effective oxygen flux reaching the corneal surface [2,3]. Previous experimental studies have demonstrated that central and peripheral oxygen transmissibility thresholds differ in their ability to prevent corneal swelling, suggesting that reliance on central measurements alone may not fully represent peripheral corneal oxygenation [6,12]. Contemporary modeling studies further confirm that total corneal oxygen consumption and regional oxygen tension are influenced by both material properties and spatial lens geometry [13,14].

Among the various techniques available for measuring oxygen permeability, the polarographic method has been widely adopted owing to its well-established theoretical foundation, reliability, and reproducibility [3]. According to ISO 18369-4, the polarographic method is defined as the reference technique for determining the oxygen permeability of contact lenses [15]. While this method is widely accepted, commercially available polarographic electrode cells are designed to measure oxygen permeability over a limited central area of the lens, typically confined to a diameter of approximately 4 mm. When measurements are performed within a restricted central region and lens power remains within a moderate range, thickness variation within the measurement area may be relatively small, allowing the central thickness to approximate the measured region [2,3]. Accordingly, the oxygen transmissibility (Dk/t) of contact lenses is conventionally calculated using the Dk value measured within this limited central region together with the corresponding central thickness.

Despite these conventions, this central, single-point measurement approach has inherent limitations because it primarily reflects the thinnest area of contact lenses. In practice, contact lenses are intentionally designed with non-uniform thickness profiles to achieve desired optical performance and wearing comfort [10,11]. Variations in lens thickness have been shown to significantly influence oxygen transmissibility and corneal oxygen delivery [2,6]. Moreover, investigations into central and peripheral oxygen transmissibility thresholds have demonstrated that peripheral corneal regions may exhibit different oxygen requirements compared with the central cornea, suggesting that central measurements alone may not fully predict hypoxic responses across the entire corneal surface [6,12].

From an anatomical and clinical perspective, the average human cornea has a diameter of approximately 11.5~12.0 mm, and the functional interaction between a contact lens and the ocular surface extends well beyond the central optical zone. Recent three-dimensional modeling studies have demonstrated that oxygen tension varies spatially across the cornea during contact lens wear as a function of material properties, thickness distribution, and lens geometry [13,14]. Even when central oxygen transmissibility is sufficient, reduced oxygen delivery in peripheral regions may induce localized hypoxic stress [5,12]. These considerations highlight the need for an oxygen permeability measurement system capable of evaluating oxygen transport over a region that more closely corresponds to the effective corneal area during actual lens wear.

In this study, we developed a modified polarographic electrode cell that enables oxygen permeability measurements over an expanded central area with a diameter of 11 mm. This measurement area closely approximates the average corneal diameter and represents an approximately 7.5-fold increase in effective measurement area compared with conventional polarographic systems. The proposed design was implemented to assess whether oxygen permeability measurements can be reliably performed over a substantially enlarged central region while maintaining methodological comparability with the ISO 18369-4 reference procedure. The performance of the modified measurement system was evaluated by comparing its accuracy, repeatability, and relative error with those obtained using a conventional polarographic electrode cell under controlled experimental conditions. Plano hydrogel contact lens samples with defined thicknesses were used to isolate the effect of measurement geometry and to ensure consistency with ISO-based oxygen permeability measurement principles.

## 2. Materials and Methods

### 2.1. Overview of the Measurement System

To evaluate the performance of the modified polarographic electrode cell, Dk measurements were conducted using both a conventional polarographic electrode cell and the newly developed electrode cell. Contact lens samples were prepared in accordance with ISO 18369-3 and ISO 18369-4 standards [15,16]. The reliability and accuracy of the Dk values obtained from the two measurement systems were compared under identical experimental conditions.

### 2.2. Design of the Modified Polarographic Electrode Cell

**Figure 1:**
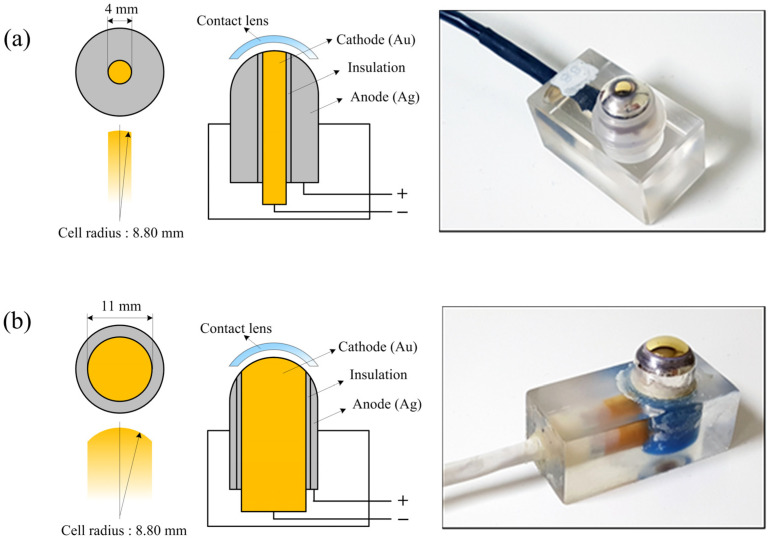
Conventional (4 mm cathode) and modified (11 mm cathode) polarographic electrode cells used for oxygen permeability measurement of contact lenses. (**a**) Conventional design; (**b**) Modified design.

In conventional polarographic electrode cells, the cathode diameter is typically limited to 4 mm, restricting the effective measurement area to a narrow central area of the lens. A modified polarographic electrode cell was designed to enable oxygen permeability measurements over a larger central area of contact lenses. Ideally, Dk measurement would be performed across the entire lens surface. However, due to the inherent limitations of polarographic measurement, maintaining stable electrical contact between the gold (Au) cathode and the silver (Ag) anode requires sufficient spacing, making full-surface Dk measurement unfeasible. In light of this constraint, and considering that the diameter of commercially available soft contact lenses is typically 14 mm or greater, the cathode diameter of the modified measurement cell in this study was expanded to 11 mm. This design not only preserves electrical stability but also enables oxygen permeability assessment over a substantially larger central area, thereby providing a more representative evaluation of oxygen transport characteristics under conditions that more closely reflect actual lens wear. As a result, the developed polarographic electrode cell allows Dk measurements over an 11 mm diameter area, corresponding to an approximately 7.5-fold increase in measurement area compared to conventional systems. The structural differences between the conventional and modified electrode cells are illustrated in Figure 1.

The modified polarographic electrode cell was designed by the research team and fabricated by a precision manufacturing company in accordance with the specified engineering and dimensional requirements.

### 2.3. Contact Lens Sample Preparation

To compare the reliability of oxygen permeability measurements obtained using the two-electrode cells, contact lens samples were fabricated with controlled thickness profiles to satisfy the testing requirements specified in ISO 18369-4 [15].

**Figure 2:**
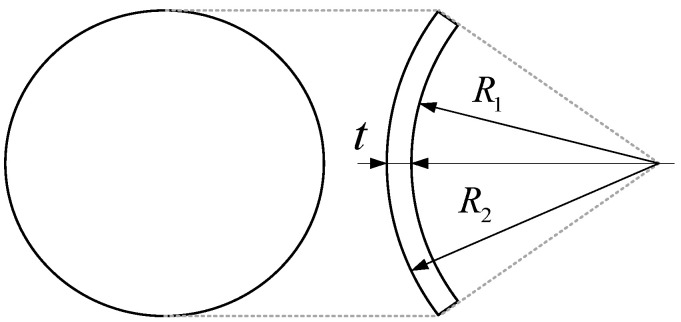
Schematic illustration of the contact lens sample geometry used for reliability comparison, showing the back surface radius (R_1_) and front surface radius (R_2_).

Four groups of hydrogel contact lenses composed of poly(2-hydroxyethyl methacrylate) (HEMA) were prepared, each with a different center thickness while maintaining uniform thickness across the lens surface. The prepared samples had center thicknesses of 60, 100, 170, and 220 μm, respectively. The back surface radius was fixed at 8.80 mm for all samples, while the front surface radius was adjusted to achieve the desired thickness. The detailed specifications of the contact lens samples are summarized in Table 1, and the lens geometry is illustrated in Figure 2.

To ensure objectivity in the measurement process, the exact Dk values associated with each contact lens sample were blinded to the experimenter.

### 2.4. Thickness Measurement

Contact lens thickness was measured using a contact-type electronic thickness gauge (Model ET-3, Rehder Development, Greenville, SC, USA).

Thickness measurements were conducted under ISO-specified environmental conditions. Prior to thickness measurement, all contact lens samples were hydrated in physiological saline solution for at least 24 h, and measurements were performed within the measurement area of the polarographic electrode cell under conditions that minimized dehydration effects. For each lens, thickness measurements were performed at a minimum of 10 randomly selected locations within the measurement area. The harmonic mean of these measurements was then used to represent the lens thickness. It should be noted that thickness measurements were not restricted to the geometric center; rather, multiple measurements were obtained across the entire measurement area of the electrode cell to account for spatial thickness variations within the evaluated region.

### 2.5. Current Measurement

#### 2.5.1. Environmental Control Conditions

Current measurements were performed under environmental conditions specified by ISO 18369-4 [15]. A temperature- and humidity-controlled chamber (TH-PE-100, JEIOTECH, Daejeon, Republic of Korea) was used to maintain stable measurement conditions throughout the experiment. The chamber temperature was controlled at 35.0 ± 0.5 °C, within the ISO-specified range (35 ± 1 °C).

Because the built-in humidity control system of the chamber was limited to 95.0 ± 0.5% relative humidity (RH), an additional passive vapor equilibration strategy was implemented to achieve near-saturated humidity conditions (≥98% RH) as required for oxygen permeability measurement. A water-filled reservoir and a water-saturated porous material were placed inside the chamber to enhance vapor saturation. The presence of condensation on the chamber walls was used as a qualitative indicator of near-saturated humidity conditions. Under these modified conditions, the chamber humidity sensor indicated relative humidity values of up to 99.8% RH.

**Figure 3:**
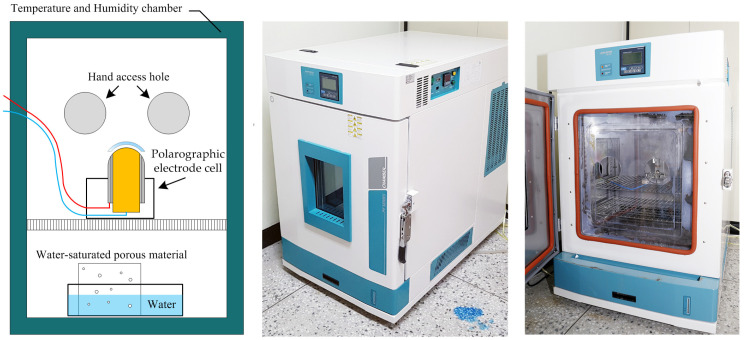
Environmental control and measurement setup for oxygen permeability (Dk) evaluation. (**Left**) Schematic illustration of the temperature- and humidity-controlled chamber showing the placement of the polarographic electrode cell and the water-saturated porous support used to maintain near-saturated humidity conditions. (**Middle**) Photograph of the chamber in the closed state to establish stable temperature and humidity prior to measurement. (**Right**) Photograph of the chamber opened for instrument operation through hand-access ports during Dk measurement.

In addition, because the chamber door must remain open during placement of the contact lens sample onto the polarographic electrode cell, a transparent insulating panel with hand access openings was fabricated and installed to minimize temperature and humidity fluctuations during sample handling. This configuration enabled stable environmental conditions throughout current measurement (Figure 3).

#### 2.5.2. Current Acquisition Procedure

The current required for the calculation of Dk was measured using a Model 201T O_2_ Permeometer™ (Createch, Greenville, SC, USA) in conjunction with the conventional and modified polarographic electrode cells. Prior to measurement, contact lens samples were hydrated in physiological saline for at least 24 h. Immediately before measurement, the samples were equilibrated in physiological saline at 35.0 ± 1.0 °C for a minimum of 2 h to ensure thermal and hydration stability. Current measurements were then performed for at least 30 min under near-saturated water vapor conditions at 35.0 ± 1.0 °C. When repeated measurements were required, the samples were rehydrated in physiological saline at 35.0 ± 1.0 °C prior to remeasurement to restore consistent hydration conditions. For each contact lens sample, at least four repeated current measurements were performed, and the harmonic mean of the measured current values was used as the representative value for subsequent Dk calculation.

### 2.6. Calculation of Oxygen Permeability

The Dk of the contact lens samples was calculated in accordance with the procedures specified in ISO 18369-4.

The Dk values were determined under the assumption that the newly developed equipment operates in an optimal state following the correct procedures, based on the principle that the boundary layer effect does not occur when the device is precisely fabricated and functioning properly [9]. As described in Section 2.4 and Section 2.5, Dk was calculated using the measured current and lens thickness obtained under controlled experimental conditions. Initially, a preliminary oxygen transmissibility value (Dk/t) was calculated from the current corrected for dark current, without applying edge-effect correction. The effective cathode area and oxygen partial pressure were taken into account according to the ISO specification, as expressed in Equation (1):

(1) $$\frac{Dk}{t}=\frac{I-I_{d}}{P_{A}\times A}\times 5.804\times 10^{-2}$$

where *I* is the measured steady-state current (A), *I_d_* is the dark current (A), *P_A_* is the oxygen partial pressure (155 mmHg), and *A* is the effective surface area of the cathode (cm^2^).

To account for edge effects arising from oxygen diffusion at the periphery of the cathode, an edge-effect correction was applied to the Dk/t values using the ISO-recommended correction method based on the model proposed by refs. [9,14,15]. This correction compensates for the influence of cathode diameter on the measured oxygen flux and enables direct comparison between the conventional electrode cell (4 mm cathode diameter) and the modified electrode cell (11 mm cathode diameter).

Finally, the final Dk value was calculated from the edge-effect-corrected Dk/t values. This calculation incorporates all measurements obtained from contact lens samples with varying thicknesses, thereby minimizing the influence of measurement variability and geometric factors.

In accordance with ISO 18369-4, the validity of the calculated Dk values was verified by ensuring that the relative deviation of Dk values obtained from repeated oxygen permeability measurements was within ±10%.

### 2.7. Comparison of Accuracy and Reliability Between the Conventional and Modified Electrode Cells

Oxygen permeability measurements were performed on the same set of contact lens samples fabricated under the conditions described in Section 2.3 using both the conventional polarographic electrode cell and the modified polarographic electrode cell. The repeatability, accuracy, and reliability of the Dk values measured by the two measurement systems were then compared. In addition, it was evaluated whether the proposed system meets the ISO acceptance criteria for newly developed measurement methods based on the relative error between the Dk values obtained using the two-electrode cells. The relative error between the two systems was calculated using Equation (2):

(2) $$Dk_{error\;rate}=\frac{\left|Dk_{c}-Dk_{n}\right|}{Dk_{c}}\times 100$$

where Dk_error rate_ represents the relative error (%) between the two measurement systems, Dk_c_ is the oxygen permeability measured using the conventional polarographic electrode cell, and Dk_n_ is the oxygen permeability measured using the modified polarographic electrode cell.

The calculated relative errors were evaluated to confirm whether the final Dk values obtained from the modified system remained within the ±10% tolerance range specified by ISO for validation of a new oxygen permeability measurement method.

The performance of the modified measurement system was evaluated in accordance with the performance requirements for alternative test methods specified in ISO 18369-4 (Clause 4.1), including repeatability and allowable tolerance criteria.

### 2.8. Data Processing and Statistical Analysis

Statistical analyses were performed using SPSS software (version 18.0, SPSS Inc., Chicago, IL, USA). Descriptive statistical analysis was conducted to summarize the measured Dk values, and paired *t*-tests were performed to compare Dk measurements obtained using the conventional and modified polarographic electrode cells. A *p*-value of less than 0.05 was considered statistically significant.

For each contact lens sample, oxygen permeability measurements were repeated multiple times under identical conditions, and the resulting Dk values were treated as paired observations for statistical comparison between the two measurement systems.

## 3. Results

### 3.1. Thickness Measurement of Contact Lens Samples

The thickness of the contact lens samples was measured under ISO-specified environmental conditions using a contact-type electronic thickness gauge (Model ET-3, Rehder Development Company, Greenville, SC, USA).

All contact lens samples were plano lenses fabricated with uniform thickness across the lens surface. Therefore, thickness measurements were performed by randomly selecting measurement positions within the maximum measurement area of the modified electrode cell (11 mm diameter). For each sample, thickness measurements were performed at least 10 times, and the harmonic mean of the resulting values was used as the representative thickness for Dk calculation in accordance with the ISO-specified method.

The measured thickness values of the contact lens samples are summarized in Table 2. The harmonic mean thicknesses for Samples A, B, C, and D were 62.52 ± 6.28 μm, 102.98 ± 1.33 μm, 176.36 ± 2.55 μm, and 221.69 ± 1.34 μm, respectively. The coefficients of variation for repeated measurements were 10.04%, 1.29%, 1.45%, and 0.60% for Samples A, B, C, and D, respectively. These results confirm that, based on the acceptance criteria commonly applied under ISO 18369 (±20% thickness variation), the samples were fabricated as precisely controlled plano contact lenses and can be regarded as flat contact lenses. This confirms that the samples provide an appropriate basis for subsequent oxygen permeability measurements and for comparative analysis between the two measurement systems.

### 3.2. Measurement of Oxygen Permeability and Reliability Comparison

To evaluate whether the modified polarographic electrode cell can replace the conventional electrode cell for measuring oxygen permeability over a larger central area, Dk measurements were performed using plano contact lens samples fabricated from the same hydrogel material (HEMA) with four different uniform thicknesses in accordance with ISO standards. The repeatability, reliability, and relative error of the Dk values obtained using the two measurement systems were compared.

The calculated oxygen permeability values measured using the conventional polarographic electrode cell (cathode diameter: 4 mm) and the modified polarographic electrode cell (cathode diameter: 11 mm) were 8.83 ± 0.12 and 8.66 ± 0.18, respectively. The coefficients of variation for repeated measurements were 1.36% for the conventional system and 2.08% for the modified system, indicating that the measurement variability of both systems remained well within the ±10% tolerance range specified by ISO.

Statistical analysis using a paired *t*-test revealed no statistically significant difference between the Dk values measured using the two electrode cells (t = 2.682, *p* = 0.055). The *p*-value of 0.055 indicates that no statistically significant difference was observed between the two measurement systems at the conventional α = 0.05 level. Therefore, the null hypothesis of no difference between the two methods cannot be rejected. Each trial reported in Table 3 represents an independent repeated current measurement performed after re-establishing steady-state environmental conditions under the ISO-specified temperature and humidity control described in Section 2.5. These results demonstrate that the modified polarographic electrode cell provides oxygen permeability measurements that are comparable in accuracy and reliability to those obtained using the conventional electrode cell, despite the substantially expanded measurement area (Table 3).

Using Equation (2), the relative error between the Dk values measured using the conventional and modified polarographic electrode cells was calculated to be 1.93%. According to ISO 18369-4, when a new measurement method is developed, the error between the new method and the reference method should be within 30% of the allowable repeatability limit (±10%) of the reference system. The calculated error rate of 1.93% therefore satisfies the ISO requirement for validation of a newly developed oxygen permeability measurement method.

**Figure 4:**
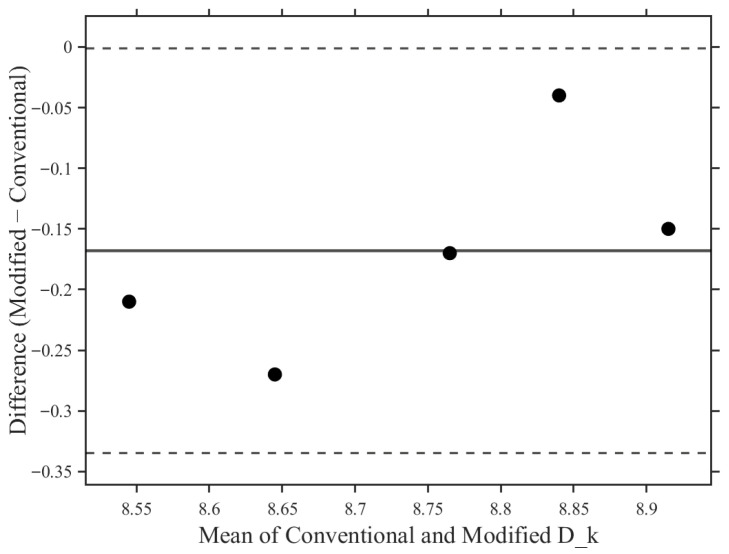
Bland–Altman plot demonstrating agreement between the conventional (4 mm cathode diameter) and modified (11 mm cathode diameter) polarographic electrode cells for oxygen permeability (Dk) measurement. The solid line represents the mean bias, and the dashed lines indicate the 95% limits of agreement.

In addition to the paired *t*-test, a Bland–Altman analysis was performed to assess agreement between the conventional and modified electrode cells. The mean bias (modified-conventional) was −0.168 (×10^−11^ cm^2^/s·(mL O_2_/mL·mmHg)), with 95% limits of agreement ranging from −0.335 to −0.001 (Figure 4). The observed limits of agreement were small relative to the overall Dk measurement range, and no evidence of proportional bias was observed across the measurement range. These findings further support the methodological comparability of the two measurement systems.

These results indicate that the modified polarographic electrode cell, which measures oxygen permeability over a larger central area with a diameter of 11 mm, provides Dk values that are consistent with those obtained using the conventional electrode cell measuring a 4 mm central area. In addition to repeatability analysis, the measurement uncertainty was estimated based on the standard deviation of repeated measurements (Type A evaluation). For the modified electrode cell (n = 5), the standard uncertainty was calculated as u = SD/√n, yielding u = 0.080 (×10^−11^ cm^2^/s·(mL O_2_/mL·mmHg)). The corresponding expanded uncertainty (k = 2) was ±0.16 (×10^−11^ cm^2^/s·(mL O_2_/mL·mmHg)), corresponding to approximately 1.8% of the mean Dk value. This magnitude of uncertainty indicates stable measurement performance despite the expanded measurement area. Taken together, these results demonstrate that expanding the effective measurement area does not introduce significant systematic deviation and maintains measurement consistency relative to the conventional configuration.

## 4. Discussion

Conventional polarographic systems are limited to measuring a narrow central area with a diameter of approximately 4 mm. This limitation suggests that, particularly in cases where lens thickness or material properties vary spatially, Dk values obtained from a narrowly defined central area may not always adequately represent the overall oxygen permeability characteristics of a contact lens.

From a methodological perspective, expanding the cathode diameter from 4 mm to 11 mm represents more than a geometric modification; it fundamentally increases the spatial representativeness of oxygen permeability assessment. The 11 mm configuration provides approximately a 7.5-fold increase in measurement area, thereby incorporating a broader distribution of local thickness variations within the functional central corneal region. This expanded spatial averaging reduces sensitivity to localized geometric irregularities and minimizes the potential dominance of the thinnest central zone in Dk determination.

The theoretical implication of this modification lies in its closer approximation to the physiological corneal diameter, which averages approximately 11.5–12.0 mm. From an experimental standpoint, the enlarged measurement area improves robustness when applied to lenses exhibiting spatial thickness gradients or heterogeneous material distributions. Therefore, even when statistical equivalence is observed under controlled ISO 18369-4 performance requirements, the expanded configuration offers enhanced practical and clinical relevance by providing a more integrated assessment of oxygen transport across the lens–cornea interface.

Compared with conventional polarographic systems limited to a narrow central measurement area with a diameter of approximately 4 mm, the modified polarographic electrode cell developed in this study enables Dk evaluation over an approximately 7.5-fold larger measurement area. The modified polarographic electrode cell demonstrated measurement performance comparable to that of the conventional electrode cell, despite the substantial expansion of the effective measurement area. The absence of a statistically significant difference in Dk values between the two systems, together with the low relative error and acceptable repeatability, indicates that reliable oxygen permeability measurements can be achieved even when the measurement area is considerably enlarged. By enabling oxygen permeability measurements over a larger central area, the proposed electrode cell offers improved representativeness of the measured Dk values. This feature is especially relevant for modern contact lens designs, in which variations in thickness profile or material distribution are often intentionally introduced to optimize mechanical or optical performance. In such cases, measurements confined to a small central area may not adequately reflect the oxygen transport characteristics actually experienced by the cornea, whereas the expanded measurement area provided by the modified measurement cell is expected to yield more representative results.

In addition to statistical hypothesis testing, the Bland–Altman analysis provided further insight into the level of agreement between the two measurement systems. The observed mean bias was small relative to the overall Dk measurement range, and the 95% limits of agreement remained within a narrow interval compared with the magnitude of the measured values. No evidence of proportional bias was detected across the evaluated measurement range. These findings indicate that the expanded measurement configuration does not introduce systematic deviation and that the modified electrode cell maintains methodological comparability with the conventional reference system. The estimated expanded uncertainty (k = 2) was approximately 1.8% of the mean Dk value, indicating that the observed bias remained within the intrinsic measurement variability of the system.

In the present study, plano lenses fabricated from a single hydrogel material (HEMA) were intentionally selected to validate the measurement system under controlled geometric and material conditions. According to ISO 18369-4, Dk is defined as an intrinsic material property and must be determined using samples with controlled thickness variations to establish a reliable thickness–current relationship. Therefore, plano lenses with uniform thickness profiles were employed to ensure strict compliance with ISO validation procedures and to isolate the effect of measurement geometry.

This design choice allowed controlled comparison of measurement geometry; however, it also represents a limitation of the present study. The findings obtained under these controlled material and thickness conditions may not fully capture the performance of the proposed system when applied to lenses with complex geometries or alternative material platforms.

We acknowledge that lenses with optical power introduce spatial thickness gradients, and lenses made from different materials, such as silicone hydrogel, exhibit distinct oxygen transport characteristics. Evaluation of such lenses may further highlight the practical and clinical advantages of wide-field oxygen permeability measurement. However, these investigations were beyond the scope of the present ISO-based system validation, which focused on methodological comparability between the conventional and modified electrode configurations.

Future studies are planned to extend this validation to powered contact lenses, cosmetic color lenses, and additional lens materials, where spatial variations in thickness and material composition may further demonstrate the broader applicability and clinical relevance of wide-field oxygen permeability assessment.

With respect to the contact lens measurement area, the modified polarographic measurement cell used in this study enabled measurements over a wider area than the conventional cell. However, due to technical limitations, measurements across the entire lens area could not be achieved. This approach was appropriate for validating the fundamental performance and reliability of the proposed measurement system; however, to fully establish its general applicability to contact lens Dk measurement, further studies are considered necessary. Specifically, technological advances are required to enable the development of instruments capable of measuring oxygen permeability across the entire area of the contact lens that contacts the cornea, or alternatively, to develop additional methods that allow estimation of full-field properties based on measurements obtained from selected areas. Moreover, further investigations should be conducted using a broader range of contact lens materials, including silicone hydrogel lenses, as well as more complex lens designs. Additional studies addressing these factors would help clarify the robustness and versatility of the modified polarographic electrode cell for oxygen permeability assessment across a wider range of contact lens products.

## 5. Conclusions

In this study, a modified polarographic electrode cell enabling wide-field Dk measurement over an expanded central area of contact lenses was developed and evaluated. Unlike conventional polarographic electrode cells, which are limited to a narrow central measurement area with a diameter of approximately 4 mm, the proposed system enables Dk evaluation over a central area with a diameter of 11 mm, corresponding to an approximately 7.5-fold increase in effective measurement area.

Experimental results demonstrated that the Dk values obtained using the modified electrode cell showed no statistically significant difference from those measured using a conventional polarographic electrode cell (t = 2.682, *p* = 0.055). The relative error between the two measurement systems was 1.93%, and Bland–Altman analysis indicated a small mean bias with narrow limits of agreement. These findings indicate that expanding the measurement area does not introduce systematic deviation and maintains methodological comparability with the conventional reference system.

The expanded measurement configuration enables oxygen permeability assessment over a region more closely aligned with the central corneal area, which may provide improved spatial representativeness compared with conventional narrow-field measurements. However, the present evaluation was conducted using a single hydrogel material (HEMA) with uniform thickness profiles. Further investigations involving additional lens materials and non-uniform thickness distributions are required to determine the broader applicability of the proposed measurement system.

Overall, the modified polarographic electrode cell provides a technically feasible approach for wide-field Dk measurement while maintaining measurement consistency with established reference procedures.

## Figures and Tables

**Table 1:** Specifications of contact lens samples designed to compare the reliability of oxygen permeability measurements obtained using two polarographic electrode cells (unit: mm).

Sample	Center Thickness	Back Surface Radius (R_1_)	Front Surface Radius (R_2_)
A	0.06	8.80	8.86
B	0.10	8.80	8.90
C	0.17	8.80	8.97
D	0.22	8.80	9.02

**Table 2:** Measured thickness of plano contact lens samples with uniform thickness (unit: μm).

Measurement	Sample A	Sample B	Sample C	Sample D
1	54	101	174	220
2	55	101	174	220
3	58	102	174	221
4	61	103	175	221
5	62	103	175	221
6	64	103	176	222
7	66	104	177	222
8	68	104	178	223
9	71	104	180	223
10	72	105	181	224
Mean	63.10	103.00	176.40	221.70
Harmonic mean	62.52	102.98	176.36	221.69
Standard deviation	6.28	1.33	2.55	1.34
Coefficient of variation (%)	10.04	1.29	1.45	0.60

**Table 3:** Calculated oxygen permeability (Dk) values measured using plano contact lens samples with uniform thickness (Dk unit: 10^−11^ cm^2^/s (mL O_2_/mL·mmHg)).

Measurement	Conventional Polarographic Electrode Cell (Cathode Diameter: 4 mm)	Modified Polarographic Electrode Cell (Cathode Diameter: 11 mm)
Trial 1	8.65	8.44
Trial 2	8.78	8.51
Trial 3	8.85	8.68
Trial 4	8.86	8.82
Trial 5	8.99	8.84
Harmonic mean	8.83	8.66
Standard deviation	0.12	0.18
Coefficient of variation (%)	1.36	2.08
Paired *t*-test	t = 2.682, *p* = 0.055	

## Data Availability

The original contributions presented in this study are included in the article. Further inquiries can be directed to the corresponding author.
